# Fascin as a useful marker for cancer-associated fibroblasts in invasive lung adenocarcinoma

**DOI:** 10.1097/MD.0000000000027162

**Published:** 2021-09-03

**Authors:** Yuko Yamada, Atsushi Kurata, Koji Fujita, Masahiko Kuroda

**Affiliations:** Department of Molecular Pathology, Tokyo Medical University, Japan.

**Keywords:** cancer-associated fibroblasts, fascin, immunohistochemistry, lung adenocarcinoma

## Abstract

Cancer-associated fibroblasts (CAFs) have been attracting attention in recent years, but their nature has not been fully elucidated. Although CAFs have been recognized as an important therapeutic target, therapeutic agents have not been developed to date. CAFs are characterized by their high migration rate and involvement in epithelial-to-mesenchymal transition with some displaying a dendritic morphology that is reminiscent of fascin expression.

The present study was designed to immunohistochemically investigate fascin expression in lung adenocarcinoma including CAFs and compare the results with existing CAF markers.

We immunohistochemically investigated fascin expression in not only cancer tissue but also CAFs from 26 autopsy cases of lung adenocarcinoma. Immunohistochemistry of α-smooth muscle actin and fibroblast activation protein was also performed.

Fascin-positive staining in CAFs was observed in all cases, with a strong correlation observed with existing CAF markers α-smooth muscle actin and fibroblast activation protein (*P* < .001). In addition, the proportion of tumor cells showing fascin-positive staining was found to correlate with its expression in CAFs (*P* < .05).

We propose that CAFs express fascin, and that fascin may mediate crosstalk between cancer tissue and CAFs. Fascin might be a novel therapeutic target for treatments that target the cancer stroma.

## 1 Introduction

Lung cancer is the leading cause of cancer-related deaths worldwide,^[1,2]^ and overcoming this disease is an urgent issue for public health. With the advent of drugs for lung cancer, such as inhibitors of epidermal growth factor receptor and immune checkpoints in recent years, the prognosis is improving.^[3,4]^ However, it is difficult to completely overcome lung cancer due to secondary mutations of driver genes and the exhaustion of immune cells. Therefore, a therapeutic agent for lung cancer based on a novel mechanism is desired.

Crosstalk with the surrounding microenvironment has a profound effect on the growth, invasion, and metastasis of lung cancer cells. It is thought that cancer cells initially occupy only about 30% of cancer tissues,^[5]^ and it has been observed empirically that cancer with a larger stroma area has a higher malignancy. Lung cancer cells proliferate surrounded by a large amount of extracellular matrix consisting mainly of collagen fibers and a stroma containing cancer-associated fibroblasts (CAFs) responsible for their synthesis. Due to presence of CAFs in the stroma, simultaneous attacking not only cancer cells but also CAFs is important for the treatment of cancer.^[5]^ However, treatments targeting CAFs have not been established, partly because immunohistochemical studies to elucidate their nature have not been conducted.

To date, the usefulness of α-smooth muscle actin (α-SMA) and fibroblast activation protein (FAP) as immunohistochemical markers for CAFs has been reported in various invasive cancers.^[6–9]^ CAFs show high migratory activity and arise from epithelial-to-mesenchymal transition^[7]^; some CAFs have a dendritic cell-like morphology.^[5]^

Fascin, a 55-kDa actin-binding protein, is one of the major regulators of the cytoskeleton and localizes in sub-membrane microspikes, filopodia, and protrusions.^[10,11]^ Fascin has been found to be a specific actin cross-linker in filopodia that provides stiffness to filopodia bundles and allows efficient coordination of actin filament elongation and bunding.^[12]^ In normal adult human tissues, fascin is expressed in nerve and dendritic cells, both cell types being characterized by having extremely large filopodia and high motility.^[10,11]^ Fascin expression in various human epithelial tumor cells has been observed, and its expression has been reported to be associated with epithelial-to-mesenchymal transition, cellular invasion, and also a poorer prognosis in various carcinomas.^[13–26]^ Nevertheless, to date, reports of fascin expression in CAFs are lacking. Since CAFs and fascin have much in common, including an association with high cellular migration rates, a dendritic morphology, and involvement in epithelial-to-mesenchymal transition, we hypothesized that fascin may be a marker for CAFs. Here we report the expression of fascin in not only cancer epithelium but also CAFs of invasive lung adenocarcinoma. From these results, it is proposed that strong fascin expression in CAFs might be a suitable novel target in cancer therapy.

## 2 Patients and methods

### 2.1 Case selection

In the present study, we used autopsy materials from 26 patients with invasive lung adenocarcinoma who were admitted to our hospital and who passed away between 1999 and 2019. The inclusion criteria were as follows: adenocarcinoma was confirmed by pathological examination; age, gender, the presence or absence of metastasis, and tumor size were known; and paraffin-embedded samples were available for specimen preparation. Whether chemotherapy and/or radiation therapy was given was not considered when selecting cases, partly because standard chemotherapy for lung adenocarcinoma has drastically changed over the last 20 years.

### 2.2 Immunohistochemistry

One representative tissue sample that included a tumor area that was key to a diagnosis was selected in each case. Tissue sections were stained with hematoxylin and eosin, along with immunohistochemical staining for fascin, FAP, and α-SMA. Immunohistochemical staining was performed, according to the manufacturer's instructions, on 4-μm sections cut from paraffin-embedded samples. After deparaffinization and rehydration, sections were autoclaved in 1 mmol/L Tris-EDTA buffer (pH 9.0) at 121°C for 10 minutes to expose antigens, cooled for 30 minutes, and endogenous peroxidases blocked by incubation in 0.3% hydrogen peroxide solution for 10 minutes. After rinsing in 0.01 mol/L phosphate buffered saline (PBS, pH 7.4), sections were incubated in blocking milk buffer (1% dry skimmed milk in PBS) for 10 minutes and incubated with affinity-purified primary antibodies for 90 minutes at room temperature. A mouse monoclonal anti-fascin antibody (clone 55K-2; dilution 1:2000; Dako, Glostrup, Denmark; M3567), a rabbit monoclonal anti-FAP antibody (dilution 1:100; Abcam, Cambridge, UK; ab53066), and a mouse monoclonal anti–α-SMA antibody (clone 1A4, dilution 1:2000; Dako; M0851) were used as primary antibodies. Samples were then washed 3 times in PBS and, thereafter, were incubated with Envision (+) Dual Link peroxidase (Dako) at room temperature for 30 minutes. 3,3-Diaminobenzidine tetrachloride was used for color development and nuclear counterstaining was performed with hematoxylin.

### 2.3 Evaluation of tissue images

Hematoxylin and eosin- and immunohistochemical-stained slides were examined by light microscope (Nikon Eclipse E400, Tokyo, Japan). Three different fields (×100) in each tissue were randomly selected from the area where cancer cells had infiltrated. Digital images of hematoxylin and eosin and immunohistochemical stainings were captured with a NY-D5000 supersystem (Microscope Network; Nikon, Tokyo, Japan) and printed on a true color printer. Positive immunohistochemical results caused the cytoplasm to appear brown. For the expression of fascin, FAP, and α-SMA in CAFs, 2 pathologists (YY and AK) independently counted the number of positive spindle-shaped elongated cells infiltrating the cancer stroma of 3 fields of view in each slide; average numbers were then calculated. For the expression of fascin in tumor cells, the percentage of positive cells was scored. Scores for the degree of stain were as follows: if positive cells accounted for 0% to 33% of all tumor cells, the score was 0; in case of 34% to 66%, the score was 1; and in case of 67% to 100%, the score was 2.

### 2.4 Statistical analyses

Clinicopathological information was allocated point scores as follows: For the patient's gender, scores were assigned as male, 1 or female, 0. Scores for poor differentiation were assigned as containing poor differentiation, 1 or not, 0. The presence of tumor metastases was scored as 1 or absence as 0. For pleural effusion, scores were assigned as a presence, 1 or absence, 0. Concerning age, for a tumor's maximal diameter (cm), a tumor's fascin positive score, and a positive CAF cell count, raw numbers were used as point scores.

Based on the point scores described above, statistical analyses using Spearman rank correlation was performed with an SPSS software package (ver17.0, Tokyo, Japan). Results were considered significant if the *P* value was less than .05.

## 3 Results

### 3.1 Clinical and histopathological characteristics

A total of 26 cases of lung adenocarcinoma, which were diagnosed and autopsied during the past 20 years at Tokyo Medical University, were identified and used in this study. The clinical and histopathological data are shown in Table 1. The cases comprised 20 males and 6 females. The mean age of patients was 70.4 ± 9.2 years. Tumor metastases were found in 20 cases, with 6 cases not showing any metastases. Of the 26 cases, pleural effusion was found in only 13 cases. The mean tumor size was 59.4 ± 40.1 mm. As for tumor differentiation, poorly differentiated components were observed in 15 cases. Out of the 26 cases, histological sub-classifications were: an acinar type, 4 cases; a papillary type, 8 cases; a micro papillary type, 1 case; and a solid type, 13 cases. Squamous cell components were found in 2 cases.

**Table 1:** Clinical and histopathological features of 26 patients.

Characteristics	Data
Mean age (years)	70.4 ± 9.2
Sex (male/female)	20/6
Tumor metastasis (+/–)	20/6
Mean tumor diameter (mm)	59.4 ± 40.1
Pleural effusion (+/–)	13/13
Poor differentiation (+/–)	15/11

### 3.2 Fascin positivity in cancer cells and CAFs along with FAP and α-SMA positivity in CAFs

**Figure 1:**
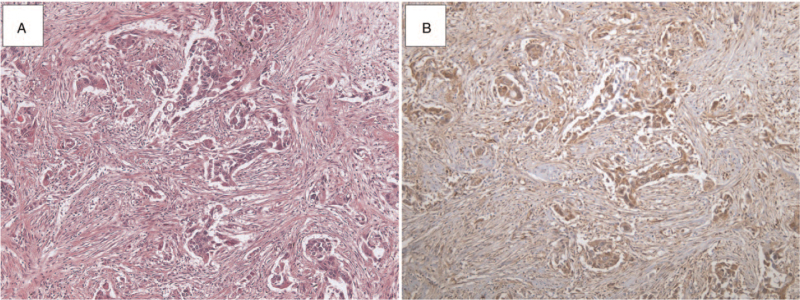
Fascin staining of lung adenocarcinoma (A) cancer-associated fibroblasts (CAFs) positive for fascin are observed as spindle-shaped elongated cells (×40). (B) Positive fascin staining in lung adenocarcinoma cells and CAFs (×40).

**Figure 2:**
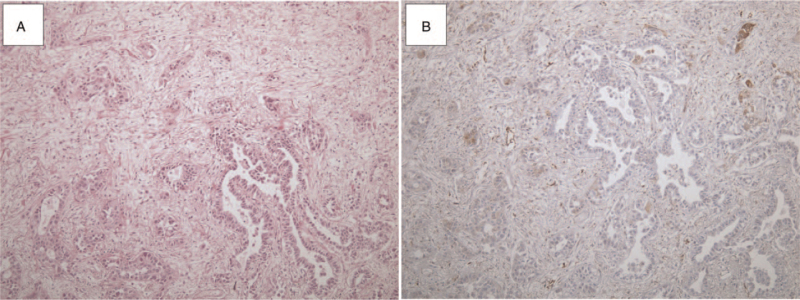
CAF markers in lung adenocarcinoma (A) adenocarcinoma and cancer-associated fibroblasts (CAFs) (×40). (B) In cases in which fascin staining was negative in tumor cells, the expression of CAF markers (smooth muscle cell actin [α-SMA], fibroblast activation protein [FAP], and fascin) was poor in CAFs (×40).

**Figure 3:**
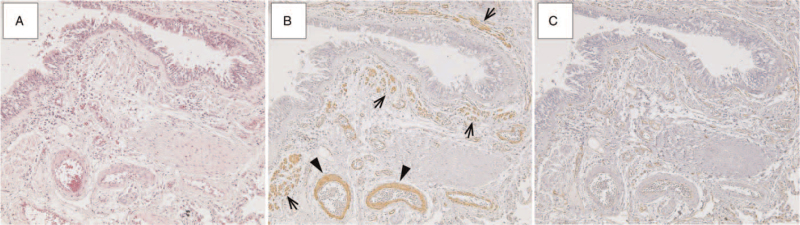
HE, α-SMA, and fascin stains in lung adenocarcinoma (A) hematoxylin and eosin (HE): bronchi and vessels in the lung are observed (×100). (B) Smooth muscle cell actin (α-SMA): positive staining in peribronchial smooth muscle (arrows) and vascular smooth muscle (arrowheads) cells (×100). (C) Fascin: fascin staining is positive only in the vascular endothelium, and negative in peribronchial and vascular smooth muscle cells (×100).

Immunohistochemical results are shown in Table 2. Positive fascin expression in tumor cells was observed in all the cases. In most cases, a large amount of tumor cells showed fascin-positivity; however, in 3 cases, only a small number of tumor cells were positive. As for CAFs that were observed as spindle-shaped elongated cells, many of them were positive for not only FAP and α-SMA, which have been used to date as CAF markers, but also fascin (Fig. 1). In many cases, the positive stainings of these 3 antibodies tended to match. The mean number of positively stained CAFs were: 40.0 ± 11.7 for fascin, 40.5 ± 10.4 for FAP, and 49.6 ± 11.7 for α-SMA in a ×100 field of view. They were often observed in places where tumors had become poorly differentiated and tumor cells had proliferated in the form of small vesicles or cords, with a strongly fibrotic interstitium surrounding them. When positive fascin staining was noted in a cancer, there was a tendency for positive fascin staining in CAFs; however, 3 exceptional cases were noted (Fig. 2). These showed occasional fascin positive staining in CAFs, even though the fascin staining of cancer cells was almost negative. As an internal control, positive α-SMA staining was found in peribronchial and vascular smooth muscle cells. However, only vascular endothelium showed positive fascin staining, while peribronchial and vascular smooth muscle cells showed negative staining (Fig. 3).

**Table 2:** Immunohistochemical results.

Characteristics	Data
Fascin (adenocarcinoma) scores (0/1/2)	3/6/17
Fascin (CAFs)	40.0 ± 11.7
FAP	40.5 ± 10.4
α-SMA	49.6 ± 11.7

α-SMA = α-smooth muscle actin, CAFs = cancer-associated fibroblasts, FAP = fibroblast activation protein.

### 3.3 Correlation analyses for clinicopathological and immunohistochemical results

Correlation coefficients were obtained (Tables 3 and 4) using these clinicopathological and immunohistochemical data. Only significant results (*P* < .05) were appointed a Spearman rho (‘r’) value, a non-parametric measure of correlation that assesses how well an arbitrary monotonic function describes the relationship between 2 variables without making any other assumptions about the particular nature of the relationship between the variables. Table 3 shows the mutual correlations within the clinicopathological data, while Table 4 shows the mutual correlations within immunohistochemical data. Our analysis found no significant correlation between clinicopathological and immunohistochemical data. However, a tendency for a positive correlation was found between cancers containing poorly differentiated components and positive fascin-staining in cancer cells (*P* = .078).

**Table 3:** Correlation analyses of clinicopathological results.

	Age	Sex	Tumor diameter	Metastasis	Pleural effusion	Poor differentiation
Age	–	NS	NS	NS	NS	NS
Sex	–	–	NS	NS	NS	NS
Tumor diameter	–	–	–	NS	NS	r = 0.614 *P* < .001
Metastasis	–	–	–	–	NS	NS
Pleural effusion	–	–	–	–	–	NS
Poor differentiation	–	–	–	–	–	–

NS = not significant.

**Table 4:** Correlation analyses of immunohistochemical results.

	Fascin (Ca)	Fascin (CAFs)	FAP	α-SMA
Fascin (Ca)	–	r = 0.422 *P*=.032	r = 0.466 *P* = .016	r = 0.504 *P *= .009
Fascin (CAFs)	–	–	r = 0.763 *P *< .001	r = 0.861 *P *< .001
FAP	–	–	–	r = 0.890 *P *< .001
α-SMA	–	–	–	–

α-SMA = α-smooth muscle cell actin, Ca = adenocarcinoma, CAFs = cancer-associated fibroblasts, FAP = fibroblast activation protein.

As shown in Table 3, a correlation existed between poor differentiation of the tumor and tumor size. This indicates that the tumor diameter increased when the tumor contained poorly differentiated components. No other significant correlation was found.

**Figure 4:**
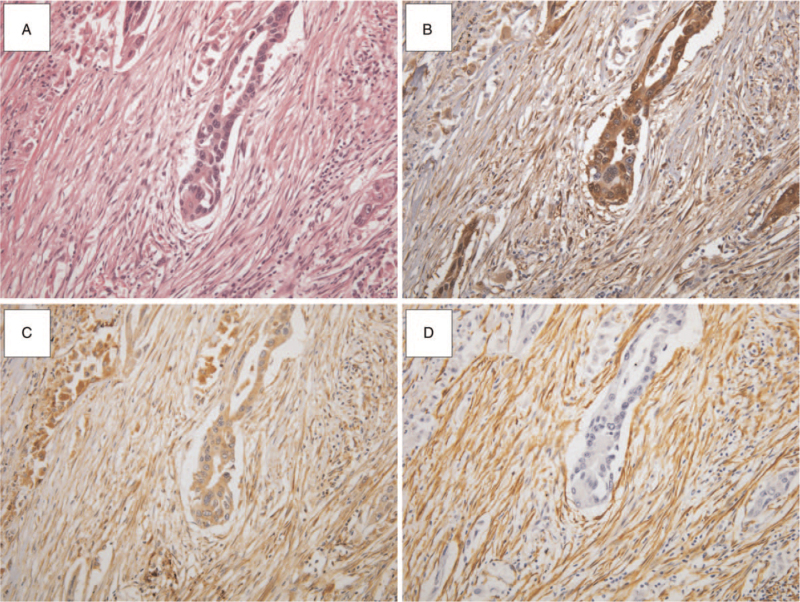
HE and CAF markers in lung adenocarcinoma (A) hematoxylin eosin (HE): adenocarcinoma and cancer-associated fibroblasts (CAFs), (B) fascin, (C) fibroblast activation protein (FAP), and (D) α-smooth muscle cell actin (α-SMA). The expression of fascin, FAP, and α-SMA in CAFs shows strong mutual correlations (*P* < .001) (×200).

As shown in Table 4, positive fascin staining in tumor cells correlated with all 3 markers, fascin, FAP, and α-SMA, staining CAFs (*P* < .05; Fig. 4). In cases where the fascin staining was negative in tumor cells, fascin, FAP, and α-SMA expression was also poor in CAFs (Fig. 2). Within CAFs, mutual correlations were found between the 3 markers: FAP, α-SMA, and fascin (*P* < .001). Fascin staining was particularly strongly correlated with α-SMA staining (*P* = .009).

## 4 Discussion

In this study, we aimed to examine the immunohistochemical properties of the CAFs of lung cancer using 26 adenocarcinoma specimens from autopsies, with a view to a long-term goal of establishing a new treatment for lung cancer targeting CAFs. To date, fascin immunohistochemistry has been extensively performed on dendritic cells and cancer tissue, but has never been conducted to identify CAFs. To the best of our knowledge, our data, for the first time, has revealed that CAFs can be immunostained for fascin, presumably associated with their extremely large filopodia and high rate of cellular migration.^[10,11]^ Since it has been reported that CAFs promote tumor progression, invasion, metastasis, and chemotherapy resistance, and are associated with a poor prognosis in pancreatic and breast cancers,^[8,27–29]^ the expression of fascin in CAFs is likely to be associated with these functions.

Clinicopathologically, a correlation existed between tumor size and poor differentiation of the tumor. That is, the diameter of the tumor increased when the tumor contained poorly differentiated components (*P* < .001). This is interpreted to mean that poorly differentiated components generally grow rapidly to a large size. Unfortunately, in our analysis, a correlation was not found between clinicopathological and immunohistochemical data. However, these results might imply that our immunohistochemical results have implications independent of clinical data.

In this study, fascin expression in cancer cells was also examined. Positive fascin staining was found in cancer tissue from all 26 specimens. Several specimens showed scant positive cells; however, about 50% or more of tumor cells were positive in 88% of total specimens. These results are similar to previous reports in which 78% of lung adenocarcinomas were fascin-positive.^[30]^ Although previous studies have shown that fascin expression correlates with a poor prognosis, such as metastasis and infiltration in breast and lung cancers,^[19,20,31]^ this study did not find a significant correlation between the presence or absence of metastasis and fascin-positive staining in tumor cells. However, cancers containing poorly differentiated components tended to show a positive correlation with fascin-positive staining in cancer cells (*P* = .078). These results also concur with those of a previous report showing that the sarcomatoid component of lung cancer was more immunoreactive for fascin.^[32]^ We postulate that the association between fascin-positive staining in cancer cells and a poorer prognosis may be a confounding factor, reflecting the fact that a poor or sarcomatoid morphology usually leads to a poorer prognosis. Alternatively, fascin-positive staining in cancer cells may promote CAFs, leading to a poorer prognosis as mentioned below. In either case, since this study is an autopsy study, it is impossible to determine the prognosis of different cancer patients with the same stage.

Furthermore, in this study, the expression of FAP, α-SMA, and fascin was investigated in CAFs. In particular, the expression of fascin in CAFs has not been reported. Herein, fascin expression was observed in CAFs, which was strongly correlated with FAP and α-SMA expression (*P* < .001). Therefore, fascin was considered to be useful as a marker for CAFs, as already reported for FAP and α-SMA stainings.^[6–9]^ As an internal control, peribronchial and vascular smooth muscle cells showed positive staining for α-SMA in the same samples; however, only vascular endothelium was found to be positive for fascin staining, as reported previously.^[14]^ This means that fascin did not co-stain smooth muscle cells in general, but only stained specific smooth muscle cells like CAFs.

Finally, we consider the implications of fascin co-expression in tumor cells and CAFs. Our results demonstrated that the proportion of fascin-positive staining in tumor cells correlates with that of FAP, α-SMA, and fascin-positive cells in CAFs (*P* *<* .05). Since fascin-positive cancer cells show higher infiltrating activity,^[19,20]^ such cells may have promoted abundant CAFs. Alternatively, CAFs may have induced cancer cells stained positive by fascin. Interestingly, the possibility of both is supported by Zhang et al^[21]^ that fascin-depleted cell lines showed reduced capacity for growth and invasion compared with wild-type cells in oral cancer, while fascin expression in cell lines was increased in cultured cells using conditioned media from CAFs. Furthermore, our previous study demonstrated that fascin-positive epithelium and surrounding fascin-positive dendritic cells correlated with thymic neoplasms.^[23]^ Thus, fascin is likely to be involved in crosstalk between tumor cells and CAFs.

This study has 2 major limitations: first, we did not consider whether chemotherapy and/or radiation therapy had been given when selecting cases, so it is unclear how chemoradiotherapy alter fascin expression; second, since this study was performed on autopsy cases only, we could not investigate prognostic impact of fascin positivity in tumor cells and CAFs.

In conclusion, we found that fascin is strongly expressed in CAFs of lung cancer. Although many studies have shown that CAFs play an important role in cancer progression, therapeutic agents against CAFs do not exist. Therefore, we believe that fascin may be a potential therapeutic target for the stroma of lung cancer. In the future, it will be important to investigate the expression of fascin in CAFs in other cancers as well.

## Acknowledgment

The authors thank Mr. Shoichiro Mineo (Department of Molecular Pathology, Tokyo Medical University) for helping us prepare specimens.

## Author contributions

**Conceptualization:** Yuko Yamada, Atsushi Kurata.

**Data curation:** Yuko Yamada, Atsushi Kurata.

**Formal analysis:** Yuko Yamada, Atsushi Kurata.

**Methodology:** Koji Fujita.

**Supervision:** Masahiko Kuroda.

**Writing – original draft:** Yuko Yamada.

**Writing – review & editing:** Atsushi Kurata, Masahiko Kuroda.
